# Enhanced weight-based clustering algorithm to provide reliable delivery for VANET safety applications

**DOI:** 10.1371/journal.pone.0214664

**Published:** 2019-04-04

**Authors:** Abubakar Bello Tambawal, Rafidah Md Noor, Rosli Salleh, Christopher Chembe, Michael Oche

**Affiliations:** 1 Faculty of Computer Science & Information Technology, University of Malaya, Kuala Lumpur, Malaysia; 2 Department of Computer Science, College of Science & Technology, Umaru Ali Shinkafi Polytechnic, Sokoto, Nigeria; 3 Centre for Mobile Cloud Computing Research (C4MCCR), Faculty of Computer Science and Information Technology, University of Malaya, Kuala Lumpur, Malaysia; 4 School of Science, Engineering & Technology, Department of Computer & Information Technology, Mulungushi University, Kabwe, Zambia; 5 Department of Computing, Faculty of Science & Technology, Kampala International University, Kampala, Uganda; University of Louisville, UNITED STATES

## Abstract

A vehicular ad hoc network (VANET) is an emerging and promising wireless technology aimed to improve traffic safety and provide comfort to road users. However, the high mobility of vehicles and frequent topology changes pose a considerable challenge to the reliable delivery of safety applications. Clustering is one of the control techniques used in VANET to make the frequent topology changes less dynamic. Nevertheless, research has shown that most of the existing clustering algorithms focus on cluster head (CH) election with very few addressing other critical issues such as cluster formation and maintenance. This has led to unstable clusters which could affect the timely delivery of safety applications. In this study, enhanced weight-based clustering algorithm (EWCA) was developed to address these challenges. We considered any vehicle moving on the same road segment with the same road ID and within the transmission range of its neighbour to be suitable for the cluster formation process. This was attributed to the fact that all safety messages are expected to be shared among the vehicles within the vicinity irrespective of their relative speedto avoid any hazardous situation. To elect a CH, we identified some metrics on the basis of the vehicle mobility information. Each vehicle was associated with a predefined weight value based on its relevance. A vehicle with the highest weight value was elected as the primary cluster head (PCH). We also introduced a secondary cluster head (SeCH) as a backup to the PCH to improve the cluster stability. SeCH took over the leadership whenever the PCH was not suitable for continuing with the leadership. The simulation results of the proposed approach showed a better performance with an increase of approximately40%– 45% in the cluster stability when compared with the existing approaches. Similarly, cluster formation messages were significantly minimized, hence reducing the communication overhead to the system and improving the reliable delivery of the safety applications.

## Introduction

A vehicular ad hoc network (VANET) is one of the promising technologies deployed to support the sharing of resources between neighbouring vehicles moving on the road in order to improve road traffic safety and provide infotainment services[1]. In VANET, vehicles communicate and exchange information through vehicle-to-vehicle (V2V) or vehicle-to-infrastructure (V2I) communication[2]. Moreover, to realize this communication vehicles are equipped with an on-board units (OBU) [3]. In the control channel all OBUs are expected to periodically broadcast their status in packets as beacons so that each OBU has real-time information of all its neighbours [4]. Dedicated short range communication (DSRC) was defined for vehicular communication which works similar to Wi-Fi; it provides a transmission range of 300– 1000m[5]. Similarly, the U.S. Federal Communication Commission (FCC) allocated 75 MHz of the spectrum band for vehicular communication. This spectrum band is divided into seven 10-MHz channels, where the control channel (CCH) is used for broadcasting safety-related messages and control information while the remaining six service channels (SCHs) are used for data transmission[6, 7].

However, the realization of the envisioned VANET applications is dependent on the reliability of the medium access control (MAC) protocol. Safety applications are the most important applications envisaged by VANET which requires reliable and timely delivery. These applications have a strict quality of service (QoS) requirement in terms of the delivery delay and the packet loss rate which cannot be guaranteed by the conventional MAC protocol particularly under heavy traffic conditions[8–10]. The high mobility of vehicles and frequent network topology changesalso affect the delivery of safety-critical applications which are time bounded particularlyin a high density network [11, 12]. To counter this problem, a clustering technique was used to ensure good coordination among the neighbouring nodes.

**Fig 1:**
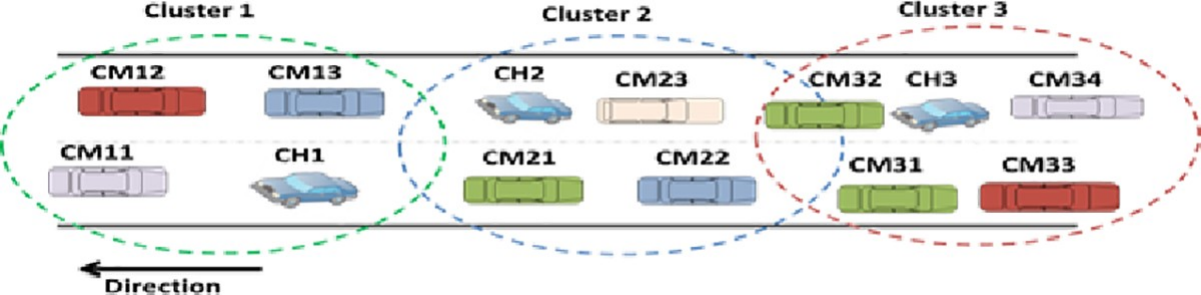
Cluster-based communication architecture.

Clustering is the process whereby a group of nodes is organized to form a sub-network on the road on the basis of some predefined metrics, which include vehicles density, velocity and geographical locations[13].This technique makes the network more robust and scalable. The cluster-based communication architecture is illustrated in Fig 1. In this technique, a cluster head (CH) is elected for each group on the basis of some defined parameters, and the remaining group members become cluster members (CMs). The elected CH bears the responsibility for coordinating the CMs and for intra-cluster communication. This generally minimizes the hidden terminal problem, thereby improving the timely delivery of safety messages[14, 15].

Furthermore, thus far, a considerable amount of research has been conducted with the aim of developing a suitable CH selection algorithm [16–21]. These algorithms attempt to minimize as less as possible the cluster reconfiguration. A good clustering algorithm requires electing a CH that can takes longer time within the cluster before it takes an exit[22]. This criterion will result in a stable cluster. The stability of a cluster is generally affected by the vehicle dynamics [23]. This is attributed to the fact that vehicles can either join the cluster or leave the cluster which can change the topology of a cluster. However, the existing cluster-based MAC approaches show that maintaining the stability of a cluster is a serious challenge because of the high mobility of vehicles and the dynamic topology changes[24]. This is because a CH can either leave or merge with another CH. It has been observed that most of the existing clustering algorithms [25–28] do not provide an appropriate mechanism to deal with a situation when the elected CH moves out of a cluster. Furthermore, this CH is responsible for coordinating all the CMs within the cluster, and if it moves out of the cluster for any reason the clustering structurewill be affected and will have to be reconfigured. Consequently, this effect will result in high communication overhead to the CCH.Additionally, this operation can lead to the loss of the channel access schedule and may lead to a transmission collision or a delivery delay of the safety messages.

In this paper, anenhanced weight-based clustering algorithm (EWCA) is proposed. Unlike the existing algorithms presented in [29, 30] where only vehicles with a similar speed range are used to form cluster groups, in theproposed approach we considered any node that was within the transmission range of its neighbours. This was done to minimize the interference among the adjacent clusters and to provide an efficient delivery of the safety messages. Similarly, to provide cluster stability and minimize the re-clustering overhead, a backup to the primary cluster head (PCH) called the secondary cluster head (SeCH) was introduced. This SeCHwas elected by the PCH considering the CM with the highest weight value. The results of our simulation were compared with the results obtained using[29, 30] to determine the effectiveness of the proposed algorithm in ensuring cluster stability and an efficient delivery of safety messages.

The rest of this paper is organized as follows. Areview of the related work is presented in Section 2. The proposed system model is presented in Section 3. In Section 4, we present the simulation and scenario setup.Theperformance evaluation based on the existing approaches is also discussed. Finally,conclusion and future work directionare presented in Section 5.

## Review of related work

This section highlights the existing clustering techniques proposed for the VANET MAC protocol in the literature. Several approaches have been used to mitigate the challenges posed by the clustering technique. In[31], the authors consider an urban scenario with several intersections. The CH selection is based on the traffic flow together with some mobility information of the vehicles. The vehicles moving in either the left or the right lane have a lower priority of becoming a CH than those moving where there is no turn at the intersection. However, this perception may sometimes result in electing a CH that could exit the cluster within a short period of time. This is because, the vehicles movementin either the left or the right lane does not necessarily implythat they will take an exit soon. The high mobility of the vehicles and frequent network topology changes affect the performance of the conventional MAC protocol and the network stability. Consequently, a stability-based clustering algorithm (SBCA) was proposed [32].The minimum velocity difference of a vehicle and the degree of neighbourhoodfor a candidate CH and its neighbourhood are used to decide which vehicle is elected as a CH. The authors also highlighted the introduction of the SeCH to improve the cluster stability, but did not specifically provide any detailed procedure for their claim. Furthermore, the vehicles direction of movement was not considered for the cluster formation process, which may result in an unstable cluster. Another significant approach considered for the clustering algorithm is the direction-aware technique [33], where the direction of movement of vehicles is prioritized for deciding which vehicle will become a CH. In this scheme, the future position of each vehicle in the cluster is predicted to control and minimize the cluster reconfiguration. Nevertheless, the periodic prediction process may add a high overhead to the communication channel and consequently reduce the MAC efficiency in implementing safety-critical applications. A region-based clustering mechanism (RCM)was also presented in[34]to improve the MAC protocol scalability. The entire network is partitioned into a number of space units where a fixed number of nodes are represented in each unit. A pool of non-interfering channels is allocated to each region or units in order to reduce the contention among the neighbouring vehicles.

The authors in[35] presented a mobilitybased clustering algorithm using affinity propagation called APROVE. In this scheme, each vehicle makes an independent decision on the basis of the message received from its neighbourhood, which includes responsibility and availability. A CH could be determined if the responsibility and availability of a node become positive. Nevertheless, using a clustering decision periodically will increase the probability of re-clustering thus, causing a high overhead to the communication channel and reduces the MAC protocol efficiency. In [29], a stability-based clustering scheme using an adaptive multi-metric algorithm was proposed. The authors used an integrated technique by combining the mobility information and the QoS metrics. Here, the vehicles compete to become suitable for being a CH in a cluster on the basis of their computed weight value. Similarly, athreshold-based clustering algorithm to form stable clusters in a highwayenvironment was presented in [30]. In this technique, the neighbouringnodes are classified as either stable or unstable on the basisof their relative speed. Vehicles are considered stable neighbours if their speed difference is less than ± Δ_th_ (predefined threshold); otherwise, they are considered unstable. Thus, only these vehicles are involved in the cluster formation process. A multi-metric algorithm is used to determine the CH giving priority to a vehicle with the highest weight value. However, both the approaches[29, 30]suggest that not all of the neighbouring nodes are suitable to be in the same cluster.This assumption can affect some neighbouring vehicles that are not suitable to be in a cluster to receive safety messages. Moreover, it can result in interference within two or more adjacent clusters as well as a less stable clustering process,consequently, affecting the reliability of the MAC protocol for an efficient delivery of the safety massages.

## System model

This section describes the design process undertaken to address the problems identified in order to meet the objectives outlined. We assumed that each vehicle was equipped with a positioning system, i.e. global positioning system (GPS), and the one-pulse-per-second (1PPS) signal for real-time synchronization. This would provide correct real-time three dimensional (3D) positions (latitude, longitude and altitude), direction and velocity. We also assumed that through a digital map each vehicle could identify its road ID. Each vehicle that joined the network will be assigned an identifier known as node id. The node id is assigned based on the vehicles’ arrival to the network beginning with zero (0). Two vehicles are considered stable neighbours if they are within transmission range of each other. Only vehicles moving in the same direction and with the same road ID will be considered to form a cluster group within a road segment on a highway. A message from neighbour vehicle moving in a different direction is not considered and should be ignored. The arrival rate of the vehicles is assumed to be a Poisson process [36].

### Metrics for CH election

In this section the different metrics considered for the CH election process are identified. These metrics involve the mobility information of each node includingthe direction of movement, roadID,meanspeedof a vehicle,nodeconnectivity level andmeandistanceof a vehicle to its neighbours. A vehicle identifies it neighbours by sharing this mobility information through periodic messages. The direction of movement of a vehicle and the road ID are only to be identified for any nearby vehicle before it can accept and process the broadcast message from its neighbours. In contrast, metrics including the mean speed of a vehicle, node connectivity level and mean distance of a vehicle are to be calculated to determine the suitability of a vehicle to become a CH. Each of thesemetrics is associated with a real value weight representing its importance. The description of thesemetrics is given below.

### Node connectivity level

Two vehicles i and j are considered neighbours if the distance between them is less than r, where r is the communication range defined by the DSRC standard. The total number of vehicles directly connected to vehicle *i* is called its connectivity level. In general, the number of neighbours of node*i* at time t in a clusteras given by [37] is calculated as follows:

$$N_{i}(t)=\sum_{j=1}^{n}dist(i,j,t)<Txrange$$ (1)

Where *j* represents the potential neighbour of vehicle *i*, *dist(i*, *j*, *t)*istrueif a connection between vehicles i and j exist at time t, otherwise it is false and *Txrange* is the transmission range of node *i*.

### Mean speed

Speed is one of the important mobility characteristics involving vehicles moving on the road. It has been widely accepted that, the speed of a vehicle is assumed to be a normal distribution [38]in a free flow traffic state;hence, the probability density function (pdf) is given in Eq (2):

$$P_{v}(v)=\frac{1}{\sigma \surd 2\pi }e^{-\frac{{\left(v-\mu \right)}^{2}}{{2\sigma }^{2}}}$$ (2)

Whereσ is the standard deviation of the vehicles’ speeds and μ is the mean speed. Each vehicle can determines how close the mean speed of its neighboursisto its current speed. Consequently, the vehicle whose speed is closest to the meanspeed of itsneighbourswillhave the highest priority of becoming a CH. The mean speed μ_veh_ of all the neighbouring vehicles is expressed in Eq (3) as follows:

$$\mu_{veh}=\sum_{j=1}^{n}\frac{\Delta d}{\Delta t}$$ (3)

Where *Δd* represents the total distance, *Δt* represents the total time covered and *j = 1* …*n* represents the neighbouring vehicles within the transmission range. However, to avoid having this value dominate and affect the result of the computation, a normalization technique wasadopted.Therefore, both the mean speed and the mean distance could be modelled using a normal distribution with the mean and variance of all the corresponding neighbouring nodes. The normalized mean speed *υ*_*normal*_ is given in Eq (4):

$$\upsilon_{normal}=\frac{v_{i}-\mu_{veh}}{\sigma_{veh}}$$ (4)

Where vehicle’s speed is represented by *v*_*i*_.

### Meandistance

Using the Euclidean distance, we calculate the mean distance ofeachvehicle that is directly linked to vehicle *i*.The shorter the distance, the faster the message will be transmitted or received by the neighbouring nodes. Each node position *n*_*p*_ can be derived from the position coordinates of the vehicles. This is represented as follows:

$$n_{p}=(x_{1},y_{1})$$ (5)

Where the position coordinates of the vehicles are represented by *x* and *y;* therefore, the mean distance μ_d_of all the vehicles could be expressed in Eq (6) as follows:

$$\mu_{d}=\frac{\sum_{j=1}^{n}\sqrt{(xi-xj{)}^{2}+(yi-yj{)}^{2}}}{N_{i}(t)}$$ (6)

The variable *j* is any neighbouringvehicle that is connected to vehicle *i*.N_*i*_*(t)*isthe total number of vehicles that are directly connected to vehicle *i* at time *t*.The normalized mean distance *d*_normal_is defined in Eq (7):

$$d_{normal}=\frac{n_{p}-\mu_{d}}{\sigma_{d}}$$ (7)

The node position is represented by n_p_, and μ_d_ represents the mean distance while σ_d_ represents the standard deviation. Each node computes its weight value ß*i* in Eq (8) to determine its suitability of becoming a CH.

$$ßi=(wf1*N_{i}(t))+(wf2*\upsilon_{normal})+(wf3*\rho_{normal})$$ (8)

Subject to:

wf1+wf2+wf3=1 (9)

Where, *wf1*, *wf2*, and *wf3*are the weight factors associated with eachparameterrespectively.The vehicle with the highest weight value ß*i*iselected as a CH.

## Cluster formation and CH election process

**Fig 2:**
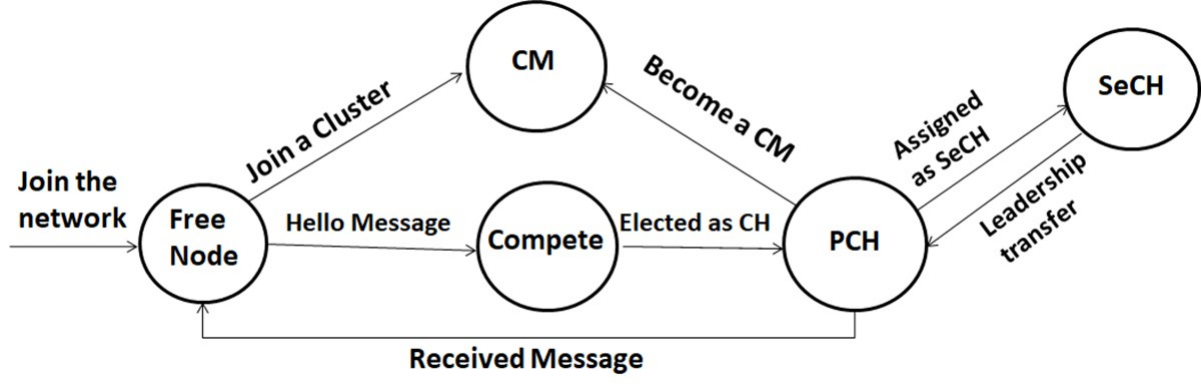
Clustering transition model.

Initially, when a vehicle joins a network,it is considered a free node and shares its current mobility information with the neighbouring vehicles within its transmission range. Similarly, it receives the same information from its nearby neighbours. Based on this received information, if a vehicle findsaPCH it will affiliate and join the existing PCH, consequently,resultingthe vehiclechanging its status to CM. In a situation when a vehicle finds more than one PCH within its transmission range, a decision needs to be taken by the vehicle to determine the most suitable PCH to join. This is achieved by comparing the position and the relative speed of the vehicle to the position and the relative speed of the available PCHs. The vehicle accepts to join the PCH if the position of the PCH is greater than the position of the vehicle. This is because, safety messages are always better to be received from the vehicle ahead, so that a decision can be taken in time to avoid the occurrence of any hazardous situation. If, at the same time, two or more PCHs are ahead of the vehicle, then the vehicle will join the PCH with a lower relative speed to the vehicle. Furthermore, if a CM loses connectivity to its PCHbecause of the vehicle’s mobility, it will scan for nearby neighbours through the exchange of periodic messages where a new cluster may be detected. In contrast, if there is no PCHnearby, the cluster formation process will be initiated.Any vehicle with the best suitable weight value within the communication range can initiate the cluster formation process, unlike the existing approaches. Each node will compete to become a CH on the basis of its computed weight valueß*i*. The mobility information of each vehicle together with the computed value is exchanged between the neighbours through periodic messages via the CCH. The node with the highest weight value is chosen as a CH. The vehicle that wins the election changes its status to PCH and is assigned a cluster ID, which will be broadcast to the entire neighbourhood. However, if two or more vehicles have the same weight value as the highest value, then the vehicle with the lowest id and having more neighbours will be elected as a CH. Each vehicle that joins the network will be assigned an identifier known as node id. The node idsare assigned on the basis of the vehicles’ arrival to the network beginning with zero (0). This will minimize the conflict of electing the PCH and the SeCH. Consequently, the elected PCH will select a vehicle from among the CMs to become the SeCH. Fig 2 showsthe proposed clustering transition model.

**Fig 3:**
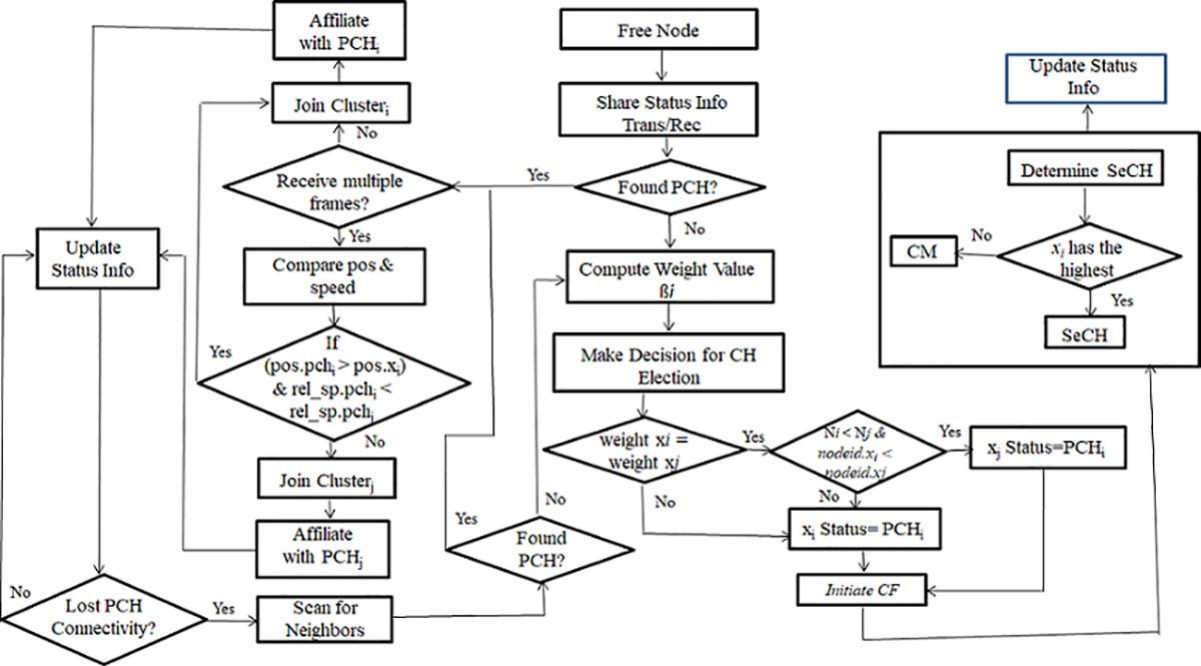
Cluster head election flow diagram.

Theflow chart in Fig 3 depicts the flow of the implementation of Algorithm 1 for the PCH/SeCH election process. The following gives the description of this algorithm.

**Algorithm 1. Cluster formation and CH election process**

**Inputs:** Number of neighbouring nodes, position, speed, road id, direction, hello message, nodeid

**Output:** Weight value

1: Broadcast. x_i_

2: **If** x_i_ receive C_jrm_**Then** // Already existing cluster within the vicinity

3:     **If** multiple beacon frames received **Then** // Determine the best suitable PCH to join

4:         **If** (pos.pch_i_>pos.x_i_) & (rel_sp.pch_i_<rel_sp.pch_j_) **Then** // Compare position and relative speed

5:             Join Cluster_i_ // Join cluster_i_ and affiliate with PCH_i_

6:             x_i_.status = CM // Vehicle acknowledges the request and joins the cluster

7:         **Else**

8:             Join Cluster_j_ // Join cluster_j_ and affiliate with PCH_j_

9:         **End If**

10:     **End If**

11: **End if**

12: Goto step 35

13: **If** x_i_ ∈ x_L_ **Then** //where x_L_is the set of all vehicles within T_range_

14:     Suitability. x_i_ () // calculate the suitability value of a vehicle

15:     x_i._Broadcast_hello_message_//vehicle broadcast status information together with suitability value

16:     T_x_. xi^←^ = = = x_i_.T_x_ () //calculate the waiting time for a cluster head response

17:     **While** T_x_. x_i_> 0 **do**

18:         **If** InitiateCluster (CH_id_) is received **Then**

19:             StopCompetition () //CH found

20:             Process InitiateCluster (CH_id_) // process the received message

14:         **Else**

21:           Decrement waiting time T_x._ x_i_

22:       **End if**

23:       **If** weight_value. x_i_ = = weight_value.x_j_ **Then**

24:         **If** (N_*i*_<N_*j*_*) & (nodeid*.*x*_*i*_*>nodeid*.*x*_*j*_*)* **Then** //determine the vehicle with more neighbours

25:             x_j._status← = = = PCH // vehicle j changes its status to PCH

26:             x_j_.id ← = = = CH_id_ // vehicle’s id is set as a cluster id

27:         **Else**

28:             x_i._status← = = = PCH // vehicle i change its status to PCH

29:             x_i_.id ← = = = CH_id_ // vehicle’s id is set as a cluster id

30:       **End if**

31:         **End if**

32:     **End while**

33:     Send InitiateCluster(CH_id_) // CH broadcasts its id to all neighbouring vehicles

34:     **End if**

35:     **End //**End cluster formation and election process

Each vehicle is assumed to maintain a list of its entire one-hop neighbour list (ONL) within its transmission range. Therefore, vehicle x_i_ builds a neighbourhood relationship by exchanging a hello message initially with the other vehicles within its communication range. If it receives a cluster join message C_jrm_, it means there is already an existing cluster within the vicinity; consequently, it will acknowledge and join the cluster. This is shown in Algorithm 1 from lines1–11. In contrast, if x_i_ does not receive C_jrm_, then there is a need for cluster formation and CH election. At this stage, each vehicle has the information about its neighbourhood, and can hence allow the computation of the suitability value to determine which vehicle will become the PCH. This new status information will be broadcast to all its neighbours’x_j_. On receiving this information, the vehicle executes the CH election algorithm to determine the highest weight value. If it has the highest value, then it will declare itself as the PCH by assigning its ID as the CH ID; otherwise it will wait for some time T_x_ for a cluster join message request. When the waiting time x_i_.T_x_ expires and the vehicle does not receive any request from any neighbouring vehicle, the vehicle initiates the cluster formation.

For a vehiclex_i_ to initiate the cluster formation as stated earlier, it has to wait for some time T_x_before accessing the wireless medium to announce its eligibility of becoming thePCH. It is known that the MAC layer is controlled by the distributed coordination function (DCF) [39] where vehicles utilize both the minimum contention window (CW_min_) and the maximum contention window size (CW_max_) values. For each unsuccessful transmission, the vehicle doubles the CW_min_ value until it reaches the max value. The period of time required for a vehicle to wait is defined in Eq 10.

$$T_{x}=\left[\frac{Nmax-\beta i}{Nmax}*(CWmax-CWmin)+CWmin\right]$$ (10)

N_max_ is the total number of vehicles within the transmission range. When two or more vehicles with the same T_x_sendsFormCluster() simultaneously, a collision will occur; as a result, none of the vehicles will be able to form a cluster. In this case, the vehicles would start new iterations of competition until one of them wins. Ifa collision still occurs, then a vehicle with the lowest id will be elected and will win the election. Subsequently, the elected vehicle will send the FormCluster() message to all the vehicles.At this time, the vehicle will change its status to PCH and set its ID as theclusterID. The procedure is shown in Algorithm 1 from lines12–35 The next step therefore is for the new PCH to determine the SeCH within the CMs. The vehicle with the highest weight value among the remaining vehicles will be elected by the PCH. The elected vehicle will be assigned the SeCH status while the remaining ones will become the CMs. This is shown in Algorithm 2 from lines 1–9. Furthermore, the description of the notations used in this paper is given in Table 1.

**Table 1:** Notations and description.

Notation	Description
CF	Cluster formation
CH	Cluster head
C_ini_	Cluster initiate message
C_jrm_	Cluster join message
CM	Cluster member
Highest_xi	Highest weight value among the cluster members
PCH	Primary cluster member
Pos	Position of a vehicle
Rel_sp	Relative speed
SeCH	Secondary cluster head
T_x_	Time to wait for a cluster head response
x_i_	Current vehicle
x_j_	Neighbour vehicle within transmission range
x_L_	Set of neighboring vehicles within transmission range

**Algorithm 2. To determine the secondary cluster head (SeCH) by the elected PCH**

**Input:** nodes weight value

**Output:** highest weight value

1: highest_ x_i_ = array [0]

2: **For** each x_i_**do** // iterate all to determine the CM to be assigned as theSeCH

3:     **If** (array [i] >highest_ x_i_**Then**

4:         highest_ x_i_ = array [i]

5:         Status. x_i_← = = = SeCH // assign the node status as SeCH

6:     **Else**

7:         Status. x_i_← = = = CM // node status assigned as cluster member

8:     **End if**

9: E**nd for**

## Cluster maintenance

In the VANET environment, vehicles can either join the highway at any time or leave because of some other reasons. However, this can affect the cluster stabilityas well asthe reliability of the MAC protocol,particularly if it involves CH. Hence, a suitable mechanism is required for maintaining the stability of the cluster. Below are the situations that trigger the maintenance procedure:

### Leadership transfer from PCH to SeCH

**Fig 4:**
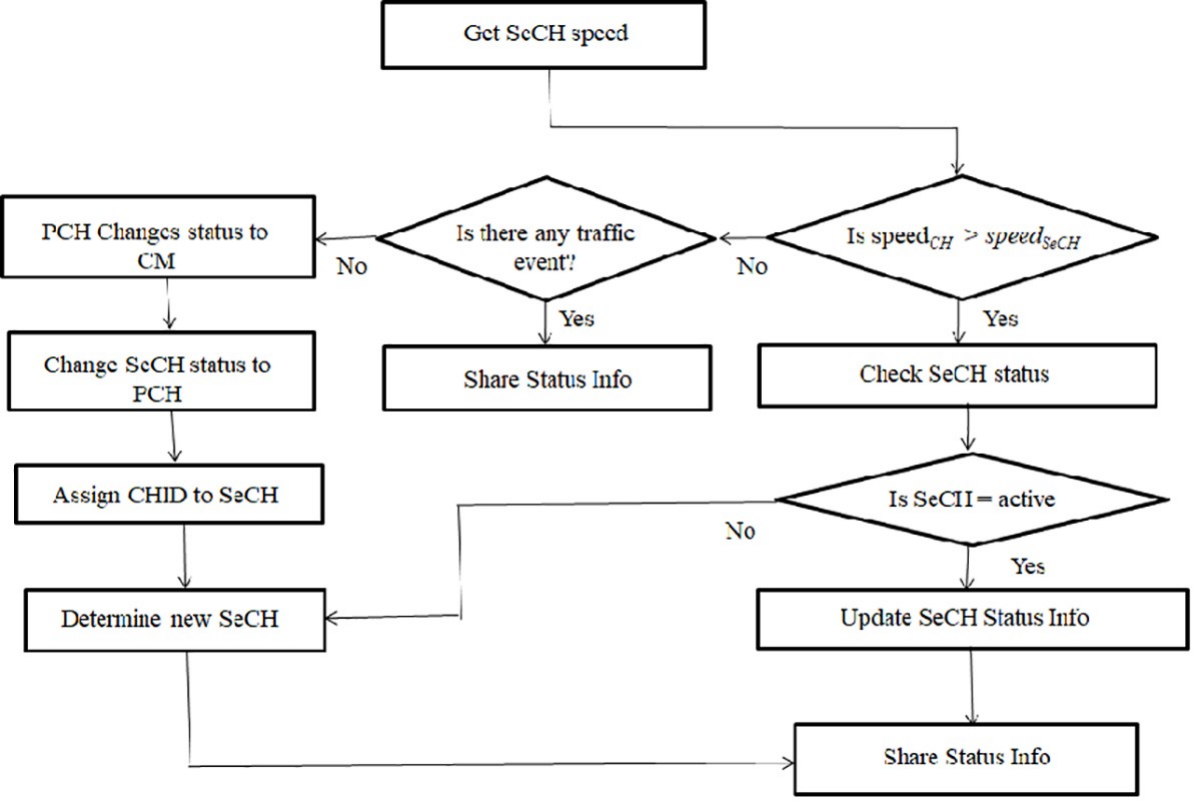
CH leadership transfer flow diagram.

When the primary CH can no longer be a CH or moves out of the cluster by taking an exit, the SeCH will take over the leadership role within the cluster instead of cluster reconfiguration. Eventually, this primary CH will change its status to a CM. This change does not affect the cluster structure, only the role of a CH will be transferred to the SeCH. The new primary CH will then select a new SeCH. Fig 4shows the flow diagram of the CH leadership transfer procedure, which is implemented in Algorithm 3.

Note that a vehicle that is about to stop by reaching its destination or wants to take an exit must slow down. Therefore, we consider speed as the main criterion to determine whether the PCH is suitable to continue with its leadership or not. Furthermore, the PCH needs to maintain the status information of its SeCH periodically in order to ensure that it is active for taking responsibility when the need arises. This is attributed to the fact that the SeCH might have left the cluster when the primary CH is trying to take an exit as well. Therefore, in the process of this update whenever it determines the current speed of the primary CH is greater, the PCH continues with its leadership. Consequently, it will update the status information of the SeCH and share this information with all the CMs.

However, in a realistic scenario, a vehicle may be forced to slow down because of some incidences that occur on the road, such as a traffic jam due to an accident or blockage on the road. In this case, the PCH will not give up its responsibility. However, when the current speed of SeCH is greater than that of PCH without any incidence happening on the road, the primary CH slows down and is about to take an exit. Hence, the primary CH resigns its responsibility of leadership and changes its status to CM. The SeCH then changes its status to PCH and assigns its ID as clusterID. A new SeCH is later determined by the new PCH using the same procedure as that in Algorithm 2 without executing the cluster formation and CH election algorithm. The updated status information is then broadcast to all the CMs within the cluster.

**Algorithm 3. Transfer of leadership from CH to SeCH**

**Input:** mean speed

**Output:** status information

1: Get speed._SeCH_ //Get the current speed of SeCHfor the periodic update

2: **If** speed._CH_ >speed._SeCH_**Then**//Check whether the current speed of CH is greater than the speed ofSeCH

3:     Broadcast_hello_message_ //Update SeCH status information

4: **Else If** any traffic incidence **Then**//Check whether there is any incidence happening on the road such as a traffic jam

5:     Update and share status information with the CMs

6: **Else**

7:     x_CH_← = = = = CM // Cluster head status changes to cluster member

8:     x_SeCH_.Status = CH //Change CH leadership to SeCH as PCH is about to take an exit

9:     x_CH_ = x_SeCH_ //Assign CID to the SeCH vehicle

10:   **End if**

11:   Assign a new SeCH and share status information with the CMs

### Cluster merging

A situation where two CHs are within the same communication range of each other requires cluster merging. In this scenario, the CH with a higher weight value will continue with the cluster leadership, while the other CH will give up its leadership. The remaining CMs will determine whether they can communicate with the new CH. Otherwise, a nearby cluster will be located or a new cluster formation process will be initiated.

### Leaving a cluster

Because of the vehicles dynamics and frequent topology changes, a vehicle may just take an exit along the highway or leave the cluster because it reaches its destination. Therefore, when a CH does not receive a periodic message from its CM in one frame cycle, it assumes that the CM is disconnected and leaves. This will result in the CH to remove the record of this CM from its list and send updated information to its current CMs.

## Simulation

In this study, the simulations were conducted using Network Simulator 3 (NS3) version 3.21. The mobility pattern of vehicles at different density was generated using a micro-traffic simulator known as Simulator of Urban Mobility (SUMO) that has realistic mobility traces. For communication over DSRC channels, we used the WAVE module that defines the IEEE802.11p standard for the PHY and MAC layers.

The scenario set up is built on 3-lanes per direction in a highway segment of 10 km. the vehicles travelled towards the same direction with a mean velocity of 30 m/s and mean deviation of 5 m/s in a free traffic flow. Additionally, the weight factor value associated with each metric for the CH election process was arbitrarily defined on the basis of the importance of each metric[37, 40]. The weight value for speed was 0.4 while that for the remaining metrics including the position and the number of neighbouring nodes was 0.3 each. The weighting factors provide the flexibility of adjusting the effective contributions of each metric. For example, in this case vehicle speed is more important and the weight value associated with speed can be made larger. The traffic density for the simulation was varied to show the different behaviour of the proposed clustering algorithm. The vehicles at low, medium and high density wereconsidered (50, 100, and 150 vehicles). To determine how the wave communication parameters affect the clustering process, we used different transmission ranges based on the different values of the tx power and the tx/rx gains at different vehicle densities. As explained in Section 3.2, once Algorithm 1 was executed, each vehicle within the vicinity of the transmission range exchanged information and cluster groups are formed. The application model for the safety application provided by [41] was modified and reused. This was done with the view of determining the delay report generated during the transmission of safety messages to the CMs. To validate the simulation results, different simulation runs were repeated for the same scenario. The simulation parameters are shown in Table 2.

**Table 2:** Simulation parameters and their values.

Parameter	Value	Parameter	Value
Simulation time	180s	DSRC channel frequency	5.9 GHz
Highway length	10000 m	Transmission rate	6 Mbps
Mean velocity	30 m/s	Message size	200 bytes
Mean deviation	5 m/s	Weight factors value	0.4, 0.3, 0.3
MAC/PHY	WAVE/IEEE802.11p	Vehicles density	50, 100, 150
DSRC channel bandwidth	10 MHz	Maximum transmission range	300 m

## Safety application scenario description

**Fig 5:**
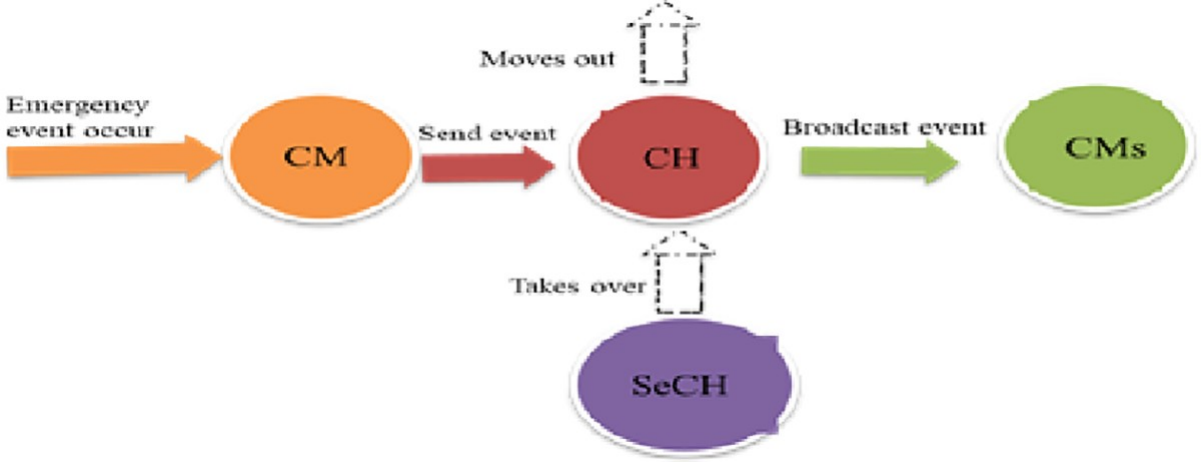
Safety application implementation scenario using proposed approach.

The aim of this scenario was to describe how the proposed clustering algorithm deals with emergency situations that occur on the road. In our simulation, the first step is to execute the clustering algorithm to form clusters and elect the CH among the contending vehicles. After forming a cluster, the status information is shared among the cluster group members, which can allow the elected CH to determine its backup node called SeCH and broadcast this updated information to all its CMs. Periodically the cluster maintenance algorithm is triggered to have a smooth transition in case the CH is about to move out of the cluster. Furthermore, each vehicle in the cluster supports the transmission of a safety message (notification or emergency event), which can be initiated randomly during the simulation. Whenever a CM detects any incident along the road such as a blind spot or a sudden break as shown in Fig 5, it will send this message to the CH with the description of the event for onward broadcast to the remaining CMs. To determine the effectiveness of the proposed clustering algorithm for the timely delivery of these event-driven messages, each CM that sends the event message will track the record of the event from when it occurred to the time when this event message was broadcast by the CH to the CMs. In a situation when an incident event occurs and the CH moves out of the cluster, the message will be either delayed or dropped before arriving at the destination. By implementing the proposed approach with the SeCH, we can minimize this challenge.

## Performance evaluation

The evaluationof the clustering process is the main focus of this analysis. A simulation was conducted to evaluate the effectiveness of the proposed technique by comparing its performance with the two existing algorithms, namely the stability-based and the threshold-based techniques presented in [29] and [30].We compared these techniques in the same environment by using the following different performance metrics: cluster stability, number of clusters formed and end-to-end delay report of emergency messages.

### Cluster stability

This metric assesses the effectiveness of the clustering algorithm in improving the stability of a cluster. The number of clusters changes as the vehicles dynamics affects the cluster stability and this depends on the clustering algorithm. Therefore, a good clustering algorithm should be able to minimize the number of times the cluster changes because of the changes in the vehicles status. CH is the node that bears all the responsibility of the management tasks within the cluster and should stay as long as possible in this state. To ensure long cluster duration and provide support for a reliable data delivery of safety applications cluster stability should be given more priority while designing the clustering algorithm. In the proposed approach, we evaluated the stability of a cluster by determining the number of times each vehicle changed its state in a cluster. This could be determined as follows:

- A CH moves out of its cluster
- A vehicle leaves and joins a nearby cluster
- Two nearby clusters merge

**Fig 6:**
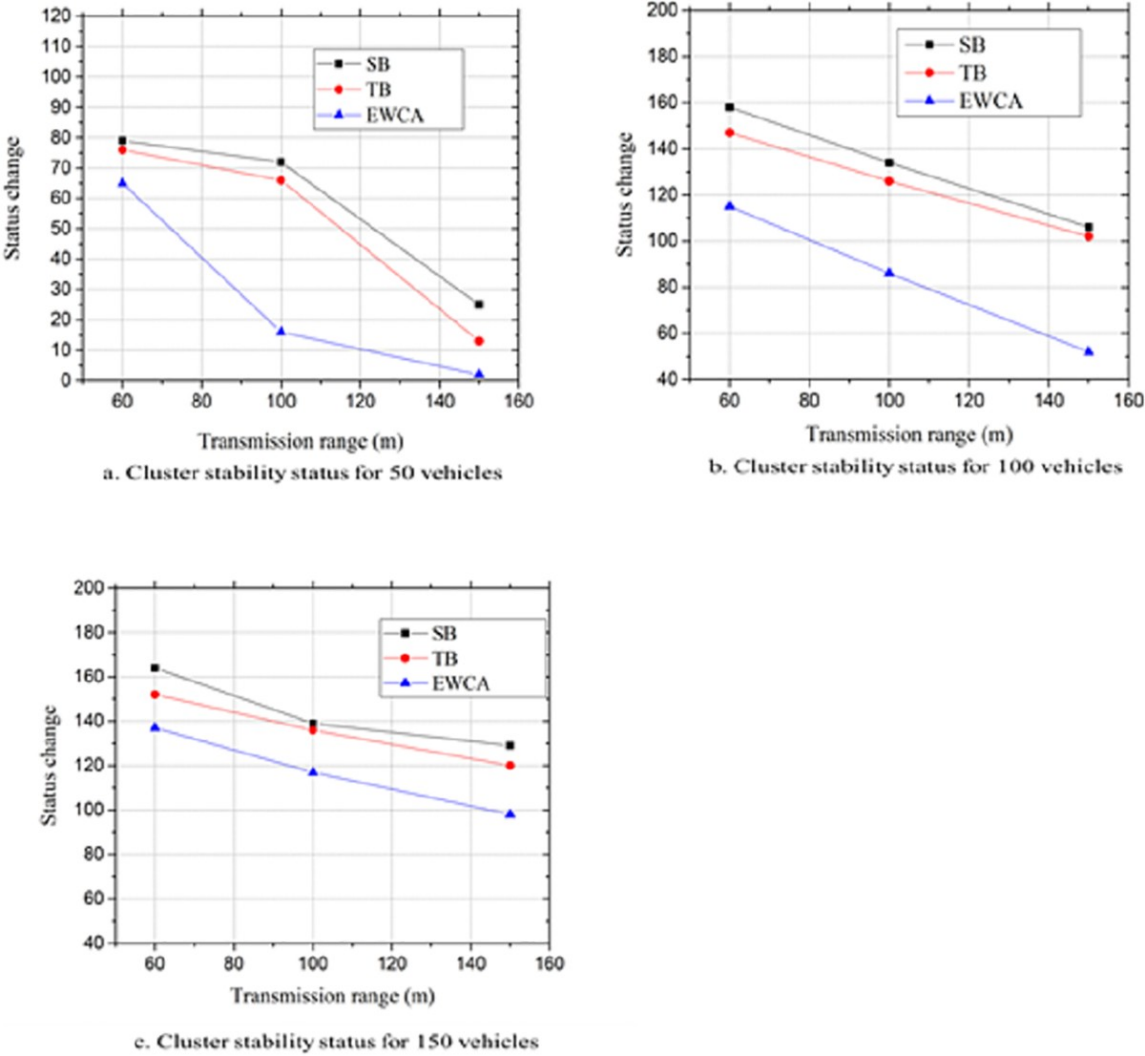
Status of a cluster at different vehicle densities.

This was tested with a varying density of vehicles in different transmission ranges as depicted in Fig 6A–6C. We deduced that from all the three different scenarios, the proposed approach EWCA performed better with fewer cluster status changesthan in the SB and TB techniques. This was attributed to the proposed procedure for cluster formation which considered any vehicle within the transmission range to be qualified to join the cluster. Secondly, the SeCH significantly minimized the possible cluster status change when the primary CH moved out of the cluster. The vehicles stayed connected with the existing cluster without the need for re-clustering or joining a nearby cluster. Consequently, the proposed approach improved the cluster stability by approximately40%– 45% as the vehicles density and transmission range increased as compared to SB and TB.

### Number of clusters formed (NCF)

**Fig 7:**
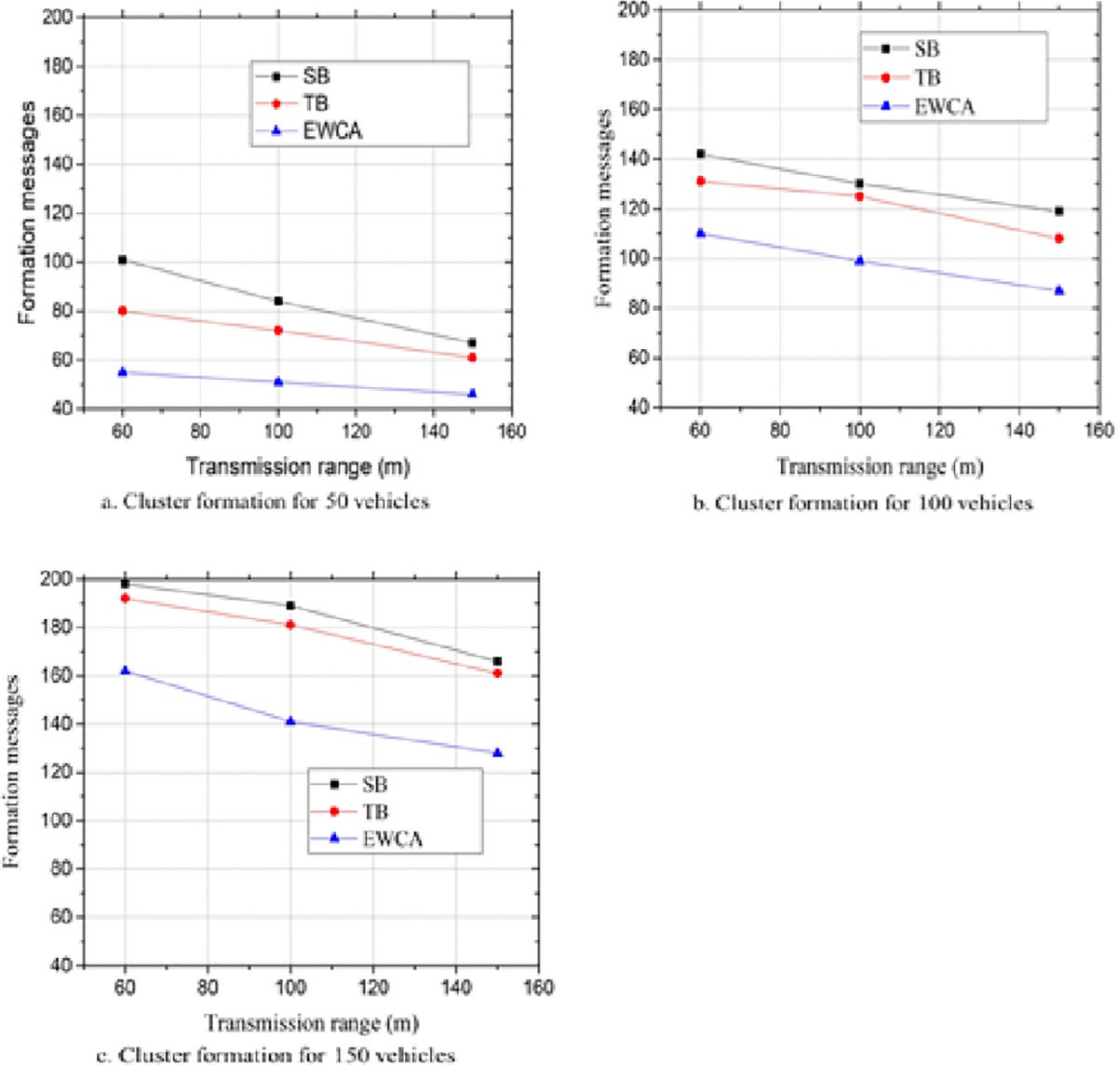
Cluster formation messages at different vehicle densities.

The total number of clusters formed is also one of the metricsused to determine the performance of the clustering algorithm. The formation of fewer clusters can reduce the network overhead. Therefore, there is a need to maintain and minimize the cluster formation rate as much as possible in order to provide a stable cluster environment. This could be achieved by minimizing the cluster formation messages. Unlike in [29, 30] where the cluster formation messages could be from either the slowest or the fastest vehicles, in the proposed approach, any vehicle could initiate the cluster formation. Fig 7A–7C show the result of the simulation. We observed that increasing the transmission range reduced the number of cluster formation messages thereby reducing the number of clusters to be formed. The proposed approach exhibited a better performance for all the different transmission ranges considered. From the graph in Fig 7, we deduced that compared with the SB approach and the TB approach, the proposed approach (EWCA) had approximately 46% and 42% less cluster formation messages respectively. This was attributed to the fact that we avoided classifying the vehicles on the basis of the speed characteristics as proposed in SB and TB. Classifying vehicles on the basis of their relative speeds will generate the formation of several clusters within the vicinity. For example, in a scenario where two or more vehicles moving in the same direction and within the same transmission range, they may fall in a different cluster if their speed difference is not within the predefined threshold. Consequently, this can significantly affect the reliable delivery of safety messages.

### End-to-end delay

**Fig 8:**
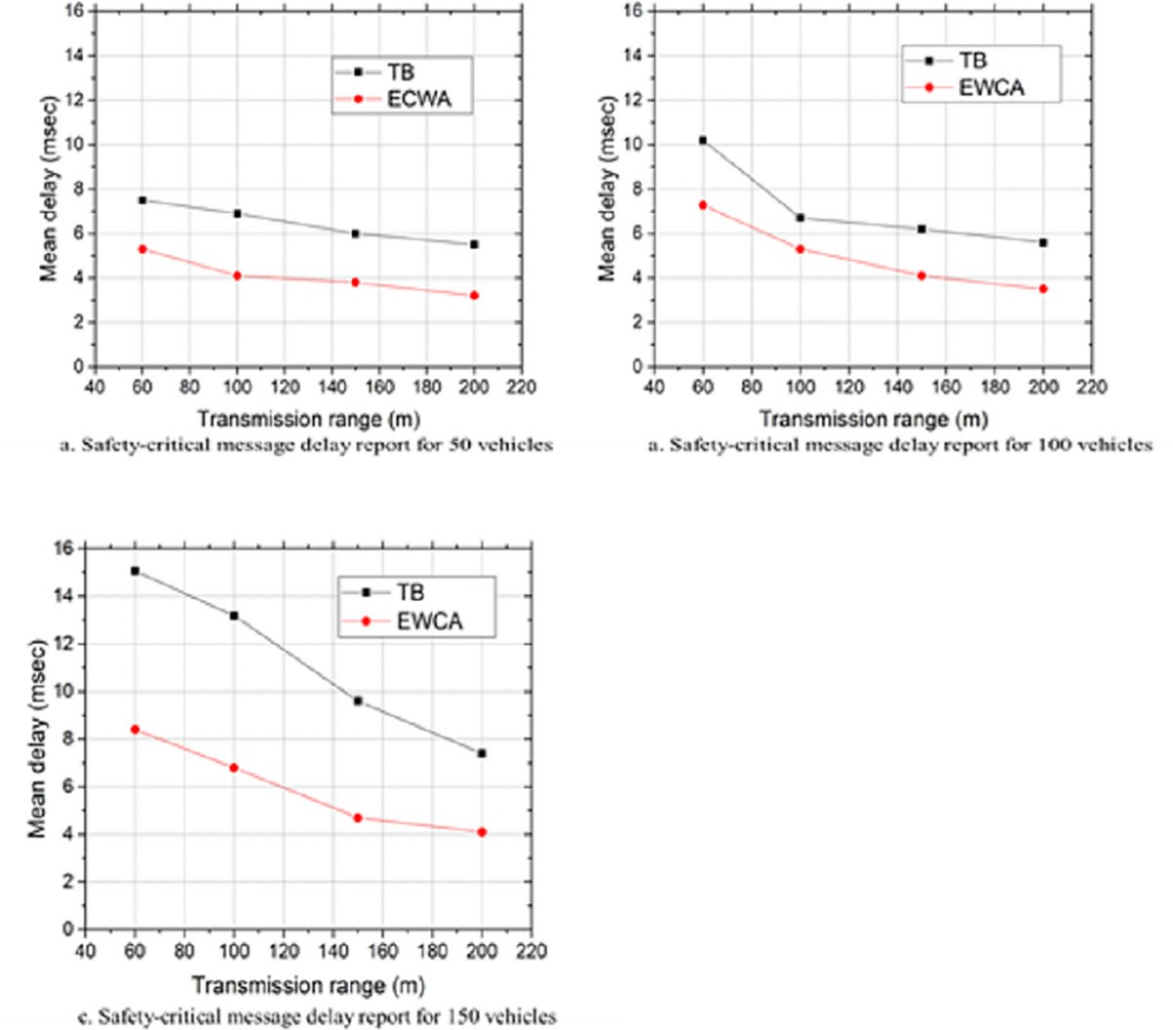
Safety-critical messages delay report at different vehicledensities.

Maintaining the same simulation parameters, in this scenario we attempted to determine the delay report of safety-critical messages particularly emergency messages because of the cluster changes. This usually happens when there is an emergency incident message from a CM to be transmitted to the CH for onward dissemination to all the CMs. However, in a situation when the CH moves out of a cluster or when cluster merges happens cluster reconfiguration will occur. Consequently, the message will be either dropped or delayed before it reaches the CMs. Fig 8, shows that the vehicles’ density and transmission range affect the broadcasting of the safety-critical messages which can result in the timely delivery of these messages. We observed that increasing the number of vehicles increases the delay but the proposed approach led to a minimal percentage increase when compared with the result obtained by using the TB approach in [30]. We found an approximately 50% reduction of the transmission delay. This was attributed to the fact that the SeCH introduced in our approach minimized the time of re-clustering whenever the CH moved out of the cluster, consequently minimizing the delay of broadcasting these safety messages to the CMs. Similarly, we deduced that increasing the transmission range could also reduce the message delivery delay.

## Conclusion and future work

Improving road safety in a VANET environment requires an efficient and reliable MAC protocol. This could be achieved using a cluster-based scheme to satisfy the stringent requirements of safety applications. In this paper, we proposed a new clustering technique based on a highway scenario. Using the mobility information of each vehicle, the clustering algorithm selects the CH on the basis of the computed weight value. To ensure and maintain cluster stability, we introduced a SeCH to take the leadership whenever the primary CH is about to leave. The speed difference of CH and SeCH was periodically checked and updated in order to ensure a smooth transition. The performance of the proposed scheme EWCA was evaluated through a simulation study. We compared the simulation results of the proposed approach with those of the two existing approaches and demonstrate a high superiority with respect to forming stable clusters and obtaining relatively few clusters. We observed that despite increasing the vehicle density and transmission range, the vehicles would still stay connected with the existing cluster for a long period of time even if the CH moved out of the cluster without the need for cluster reconfiguration. Our approach improved cluster stability by approximately40%– 45% as compared to SB and TB. Furthermore, by using the worst-case scenario,we significantly minimized the delay in broadcasting safety messages. When compared with the threshold based approach, the proposed approach achieved an approximately 50% reduction of the delay in broadcasting safety messages to the CMs by CH. This was achieved because of the introduction of SeCH, which minimizedthe re-clustering problem. Note that CH has the responsibility of allocating a slice of time to its CMs in a cluster-based TDMA MAC protocol. With this notion, in our future work, we intend to implement a dynamic slot allocation scheme to this clustering process in order to minimize the rate of merging collision problem among the adjacent clusters. This may enhance the reliable delivery of the safety applications within a vehicular environment.
